# TRIM6 affects the prognosis of acute myeloid leukemia through the PI3K/AKT signaling pathway and is associated with immune infiltration

**DOI:** 10.1371/journal.pone.0329560

**Published:** 2025-09-17

**Authors:** Jingxin Zhang, Wu Zhou, Yuancheng Liu, Chenglong Hu, Siqi Wang, Jianxia Xu, Sixi Wei

**Affiliations:** 1 Center for Clinical Laboratories, The Affiliated Hospital of Guizhou Medical University, Guiyang, Guizhou, China; 2 Department of Clinical Biochemistry, School of Clinical Laboratory Science, Guizhou Medical University, Guiyang, Guizhou, China; European Institute of Oncology, ITALY

## Abstract

**Objective:**

Acute Myeloid Leukemia (AML) is a heterogeneous disease with limited therapeutic options and poor prognosis for patients. Therefore, the aim of this study was to evaluate the potential value of TRIM6 as a prognostic marker in AML.

**Methods:**

In this study, the low-expressed gene TRIM6 was obtained by analysis of The Cancer Genome Atlas (TCGA-AML) and Genotype-Tissue Expression (GTEx) databases.The prognostic impact of TRIM6 was analyzed using Kaplan-Meier curves, univariate COX, multivariate COX, and columnar plot models. The signaling pathways associated with TRIM6 were obtained using Gene Set Variation Analysis (GSVA) and Gene Set Enrichment Analysis (GSEA) methods. Infiltrating immune cells associated with TRIM6 expression were analyzed using the “CIBERSORT” method. Mutations in AML patients were characterized using relevant Single Nucleotide Polymorphism (SNP) data. The effect of TRIM6 expression on AML cell progression was further analyzed by qRT-PCR, CCK-8 assay, flow cytometry and Western blot.

**Results:**

By analyzing TCGA-AML and GTEx data, TRIM6 was found to be under-expressed in AML patients, and Kaplan-Meier curves, one-way and multifactorial Cox regression models suggested that under-expression of TRIM6 had a poor prognosis. In addition, monocyte, M2 macrophage and memory B cell infiltration levels were found to be higher in the TRIM6 low-expression group than in the TRIM6 high-expression group based on the “CIBERSORT” method. Further mutational characterization showed that TRIM6 expression was positively correlated with NPM1 mutations and negatively correlated with mutations in RUNX1, TP53 and ASXL1. Enrichment analysis revealed that TRIM6 expression was associated with the PI3K/AKT signaling pathway and immune response. In addition, in vivo and in vitro experiments further demonstrated that TRIM6 expression could inhibit AML progression via PI3K/AKT.

**Conclusion:**

TRIM6 is expressed at low levels in AML, correlates with immune infiltration, and may affect AML progression through the PI3K/AKT signaling pathway.

## 1. Introduction

Acute myeloid leukemia (AML) is a hematologic malignancy characterized by the rapid, uncontrolled proliferation of myeloid precursor cells in the bone marrow and blood. The annual incidence of AML is approximately 4.3 per 100,000, with a median onset age of 68 years, which increases with age [1]. The pathogenesis of AML is primarily driven by genetic mutations and immune dysfunction [2]. The tumor microenvironment (TME) has emerged as a key factor in the progression and pathogenesis of AML [3,4]. Cytogenetic markers, including FLT3, NPM1, TP53, CEBPA, IDH, DNMT3A, and KIT mutations, are vital tools for risk stratification in AML, guiding personalized treatment plans and improving patient prognosis [5]. Consequently, identifying novel prognostic biomarkers is essential for enhancing patient monitoring and outcomes.

The tripartite motif-containing (TRIM) protein family, composed of over 70 members, is characterized by a conserved structure comprising a RING finger domain, one or two B-box domains, and a coiled-coil region. These proteins are involved in a wide range of biological processes, including innate immune regulation, cell proliferation, autophagy, viral replication, and the development and progression of malignancies [6–9]. In recent years, accumulating evidence has demonstrated that several TRIM family members play critical roles in AML. For example, TRIM25 is upregulated in AML and promotes the proliferation and migration of leukemic cells [10]; TRIM31 has been reported to enhance cell proliferation and induce apoptosis, as well as regulate chemoresistance in AML cells, partially via the Wnt/β-catenin signaling pathway [11]. These studies suggest that TRIM proteins are closely involved in the pathogenesis of AML and may serve as potential therapeutic targets or prognostic biomarkers.

TRIM6, a member of the TRIM family, possesses E3 ubiquitin ligase activity [12]. It is highly expressed in mouse embryonic stem cells, where it interacts with Myc to inhibit its transcriptional activity, thereby maintaining stem cell pluripotency [13]. Moreover, TRIM6 facilitates the ubiquitination of key proteins to promote the replication of viruses such as Ebola virus and SARS-CoV-2 [14,15]. TRIM6 has also been implicated in cardiomyocyte apoptosis and aggravates myocardial ischemia/reperfusion injury [12]. Recent studies have suggested its prognostic value in several solid tumors, including gliomas [16] and hepatocellular carcinoma [17]. However, despite growing insights into the biological roles of TRIM6 in systemic diseases and solid tumors, its expression profile, functional mechanisms, and prognostic relevance in AML remain largely unexplored, representing a significant gap in current research.

Therefore, this study aimed to systematically analyze the expression pattern of TRIM6 in AML using data from The Cancer Genome Atlas (TCGA-AML) and the Genotype-Tissue Expression (GTEx) databases. Through a combination of bioinformatics prediction and in vitro experimental validation, we investigated the potential role of TRIM6 in the pathogenesis and prognosis of AML, with the goal of providing new insights into molecular biomarkers for this disease.

## 2. Materials and methods

### 2.1 Differential expression analysis of TRIM6

RNA-seq data and corresponding clinical information for AML were obtained from the TCGA database (https://cancergenome.nih.gov/), encompassing 151 AML samples. Seventy control samples were downloaded from the GTEx database (https://www.gtexportal.org/home/)for differential expression analysis of TRIM6 after merging and adjusting the data with TCGA. Data preprocessing, including normalization and log-transformation, was performed using R (v4.1.3).

### 2.2 Construction and validation of prognostic columnar plots for TRIM6

To establish a prognostic model for predicting overall survival (OS) in patients with AML, a nomogram was constructed based on age, gender, and TRIM6 expression levels. Calibration curves for 1- and 3-year survival were then plotted to assess the consistency of the OS predictions. Univariate and multivariate Cox regression analyses were conducted to further validate TRIM6 as a significant clinical predictor of OS [18].

### 2.3 Co-expression analysis of TRIM6

Co-expression analysis of TRIM6 in AML was conducted using RNA-seq data from the TCGA database. Genes with a correlation coefficient of 0.5 and a p-value of less than 0.05 were selected [19]. A correlation analysis plot and heatmap were generated to visualize the most significantly co-expressed genes with TRIM6.

### 2.4 Gene set difference analysis and gene set enrichment analysis

Gene Set Variation Analysis (GSVA) was employed to determine the biological functions of the samples by converting the gene expression matrix into a gene set expression matrix, allowing for the analysis of pathway-level changes [20].

Based on TRIM6 expression levels, patients were divided into high- and low-expression groups. Gene Set Enrichment Analysis (GSEA) was used to identify pathway differences between these groups. Annotated gene sets from the MsigDB database (v7.0) were selected as the background for pathway enrichment analysis. Gene sets with significant enrichment (adjusted p-value < 0.05) were ranked, and consistency scores were calculated to explore the impact of TRIM6 expression on signaling pathway alterations.

### 2.5 Correlation analysis between TRIM6 and immune cell infiltration

To evaluate the correlation between TRIM6 expression and immune cell infiltration in AML, the “CIBERSORT” algorithm was applied to analyze RNA-seq data from patients with AML. This dataset included 547 biomarkers and was used to infer the relative proportions of 22 immune cell types [21]. Differences in immune cell infiltration between high and low TRIM6 expression groups were analyzed and visualized using box-and-whisker plots. The correlation between TRIM6 expression and immune cell infiltration was further represented by a lollipop plot.

### 2.6 Correlation analysis of drug sensitivity of TRIM6

Pharmacogenomic predictions were made based on the largest pharmacogenomics database,Genomics of Drug Sensitivity in Cancer (GDSC v1; https://www.cancerrxgene.org/), to estimate thehalf maximal inhibitory concentration (IC50) values of TRIM6 expression in response to common chemotherapeutic agents, such as paclitaxel, bortezomib, and erlotinib.The analysis was conducted using the R package pRRophetic(v0.5), a tool designed to predict tumor drug sensitivity by establishing predictive models that correlate baseline gene expression levels with in vitro drug responses observed in cancer cell lines, wherein the algorithm employs ridge regression to estimate clinical chemotherapeutic outcomes.

### 2.7 Key miRNA prediction of TRIM6

TRIM6-associated miRNAs were identified through reverse prediction using the miRcode database (v11; accessed April 9, 2024) with its default integrated prediction algorithm. Definition of TRIM6-associated miRNAs employed the database’s default prediction score threshold. The resulting miRNA-gene network was visualized using Cytoscape software (v3.7.1) [22].

### 2.8 Reagents and instruments

Fetal bovine serum was purchased from Shanghai Gitai Ecosci Biotechnology Co., Ltd. (Shanghai, China); RPMI 1640 medium was obtained from Gibco (USA); penicillin, streptomycin, and red blood cell lysis buffer were purchased from Solarbio Life Sciences (Beijing, China). Reverse transcription reagents and qRT-PCR kits were sourced from TaKaRa (USA), and the CCK-8 assay kit was purchased from MedChemExpress (USA). The Annexin V-APC/Cyanine7/7-AAD cell apoptosis detection kit was purchased from Procell (Wuhan, China), and the cell cycle detection kit was obtained from Shanghai Qihai Fuqin Biotechnology Co., Ltd. (Shanghai, China). PI3K and P-PI3K antibodies were sourced from Abimatrix Pharmaceutical Technology Co., Ltd. GAPDH, AKT, P-AKT, PCNA, BAX, Bcl-2, goat anti-mouse secondary antibody, and goat anti-rabbit secondary antibody were purchased from Proteintech (Wuhan, China). ECL chemiluminescence reagent was obtained from Millipore (USA). The TRIM6 overexpression plasmid was purchased from Guangzhou Aiqi Biotech Co. The PCR instrument, Nanodrop One spectrophotometer, enzyme marker, and Applied Biosystems QuantStudio 5 real-time fluorescence PCR instrument were purchased from Thermo Fisher (USA). The FACSCanto II flow cytometer was obtained from BD (USA), and the gel imaging analyzer was sourced from Bio-Rad (USA).

### 2.9 Sample collection

Between 01/01/2024 and 31/12/2024, the Affiliated Hospital of Guizhou Medical University in Guizhou, China, collected blood samples from 33 AML patients and 30 healthy donors.The hospital’s Ethics Committee approved the collection and use of these samples, and all participants gave written informed consent. This part of the study is a retrospective one, and during and after data collection, the authors couldn’t obtain any information that could identify participants. Whole blood samples were treated with ammonium chloride buffer to lyse red blood cells, and the remaining leukocyte fraction was used for total RNA extraction. RNA extraction was performed following the standard protocol outlined in the TRIzol reagent manual.

### 2.10 Cell culture

MOLM-13 and Kasumi-1 cells, obtained from the Cell Bank of the Chinese Academy of Sciences (Shanghai, China), were cultured in RPMI-1640 complete medium supplemented with 10% fetal bovine serum and 1% penicillin-streptomycin. The cells were incubated at 37°C with 5% CO2, with media changes and passaging every 2–5 days.

### 2.11 RNA extraction and qRT-PCR to detect TRIM6 expression

Total RNA from clinical samples, MOLM-13, and Kasumi-1 cells was extracted using TRIzol™ reagent and reverse transcribed into cDNA with a commercial kit. Real-time amplification was carried out using qRT-PCR reagents, and the mRNA expression of TRIM6 was quantified by real-time fluorescence PCR (qRT-PCR). GAPDH served as the internal reference gene, with primers F: 5’- TGGCGCTGAGTACGTCG-3’ and R: 5’- ACACCCATGACGAACATG-3’. The primers for TRIM6 were F: 5’- AGTACCAGCCTCATAATCTAAG-3’ and R: 5’- TTCCCCTTCCCTGCTTCTCTCT-3’. The amplification program was as follows: 95°C for 3 min, 95°C for 10 s, 60°C for 30 s, 60°C for 5 min, and 40 cycles. Relative gene expression was calculated using the 2^-ΔΔCt^ method.

### 2.12 Construction of stable overexpression cell lines

TRIM6 gene overexpression (OE-TRIM6) plasmid and control (Vector) plasmid were mixed with transfection reagents and introduced into 293T cells. Supernatants were collected at 24, 48, and 72 hours, concentrated, and used to infect MOLM-13 and Kasumi-1 cells. GFP expression was observed under fluorescence microscopy at 72 hours post-transfection. The transfected cells were cultured in a complete medium with puromycin (1 μg/ml), maintained in a 37°C incubator with 5% CO2, and the medium was changed every 2–3 days. Selection was continued for 7 days. Overexpression efficiency was validated by qRT-PCR and Western blot.

### 2.13 Western blot determination of related proteins

For protein analysis, cell precipitates were collected and lysed using RIPA buffer (100:1 lysate to protein phosphatase inhibitor). The cells were sonicated, centrifuged at 12000 rpm for 20 minutes at 4°C, and the supernatant was collected. Protein concentrations were measured using a BCA kit, and 35 μg of protein was loaded for SDS-PAGE electrophoresis. The protein was then transferred to a PVDF membrane, blocked with 5% skimmed milk at room temperature for 2 hours, and incubated overnight at 4°C with the primary antibody. After incubation with the secondary antibody for 1 hour at room temperature, the protein bands were detected using ECL chemiluminescence. The grayscale of the bands was analyzed using ImageJ software, and relative protein expression levels were normalized to GAPDH.

### 2.14 CCK-8 detection of AML cell proliferation

Cell proliferation ability of AML cells was assessed using the CCK-8. Overexpressing TRIM6 cells and control cells were seeded in 96-well plates at a density of 1.2 × 10^4^ cells per well. At 0, 24, 48, and 72 hours, 10 µL of CCK-8 reagent was added to each well. After 2 hours of incubation, the absorbance was measured at 450 nm using an enzyme reader. The experiment was repeated three times, with three replicates per condition.

### 2.15 Detection of cell cycle and apoptosis by flow cytometry

For cell cycle analysis, cells were plated in 6 cm dishes.l dishes at a density of 1 × 10^6^ cells per well. After 48 hours, the cells were washed twice with ice-cold PBS, fixed with 1 mL of 70% ethanol, and incubated overnight at 4°C. The cells were then resuspended in cold PBS and centrifuged. Propidium iodide (PI) staining was performed at a concentration of 50 µg/mL. The cells were incubated in the dark at 37°C for 30 minutes, then analyzed by flow cytometry (FACSCanto II) using an excitation wavelength of 488 nm to detect red fluorescence. The cell cycle distribution was analyzed with NovoExpress software. Each experiment was repeated three times.

For apoptosis detection, cells were seeded in 6-well plates at a density of 5 × 10^5^ cells per well. After 48 hours, cells were washed twice with ice-cold PBS, resuspended in 100 µL of 1 × Binding Buffer, and stained with 5 µL of Annexin V-APC and 5 µL of 7-ADD. After a 15-minute incubation at room temperature in the dark, apoptosis was analyzed by flow cytometry (FACSCanto II). Apoptosis rates were quantified using NovoExpress software. Each experiment was repeated three times.

### 2.16 Statistical analysis

All data are presented as mean ± standard deviation (SD). Statistical analysis was performed using GraphPad Prism 9.5 (GraphPad Software Inc., CA, USA). For comparisons between two groups, a t-test was used, and for comparisons between more than two groups, one-way analysis of variance (ANOVA) was applied.The log-rank test was used for survival analyses, and Pearson’s correlation coefficient was used to assess linear correlations between variables. Statistical significance was defined as p < 0.05 (*p < 0.05, **p < 0.01, ***p < 0.001).

## 3. Results

### 3.1 TRIM6 is lowly expressed in patients with AML and associated with poor prognosis

**Fig 1:**
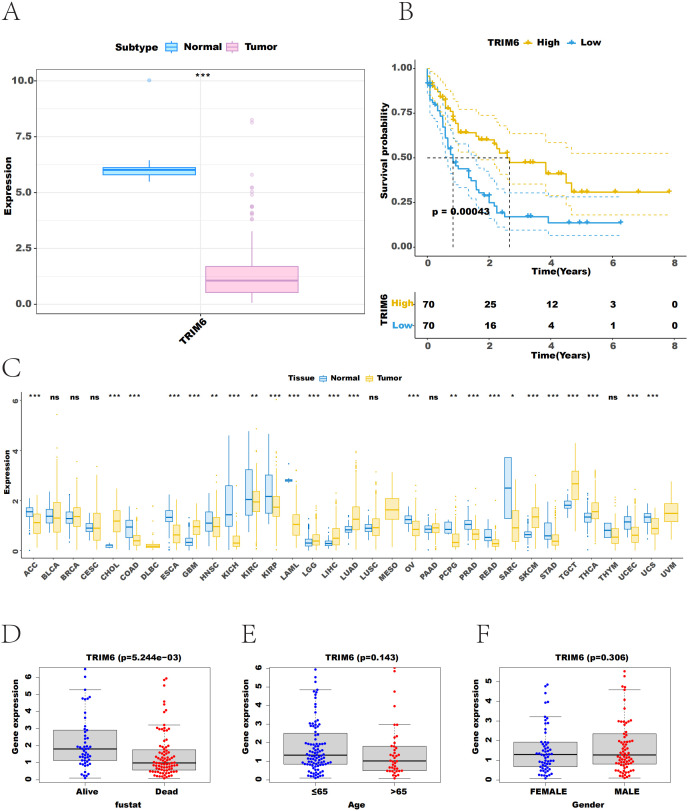
TRIM6 is lowly expressed in patients with AML and associated with poor prognosis. (A) Differential expression of TRIM6, with blue representing control patients and pink representing tumor individuals. (B) Comparison of TRIM6 expression between cancer and control samples from TCGA and GTEx databases, with yellow representing tumor samples and blue representing control samples. (C) Kaplan-Meier survival curves for overall survival of patients with AML based on TRIM6 expression. (D-F) Correlation between TRIM6 expression and clinical indicators.*p < 0.05, **p < 0.01, ***p < 0.001.

The mRNA expression data of patients with AML were retrieved from the TCGA database and integrated with GTEx data. The results showed that TRIM6 expression was significantly reduced in tumor samples compared to control samples (Fig 1A and 1B). Survival analysis revealed a p-value of 0.00043 for TRIM6, indicating that patients with high TRIM6 expression had significantly longer OS compared to those with low expression (Fig 1C). Additionally, TRIM6 expression was found to be significantly associated with the Fustat staging of the patients (Fig 1D–1F). These results suggest that down-regulation of TRIM6 in patients with AML correlates with poor prognosis.

### 3.2 Enrichment analysis of related genes and pathways of TRIM6 in AML

**Fig 2:**
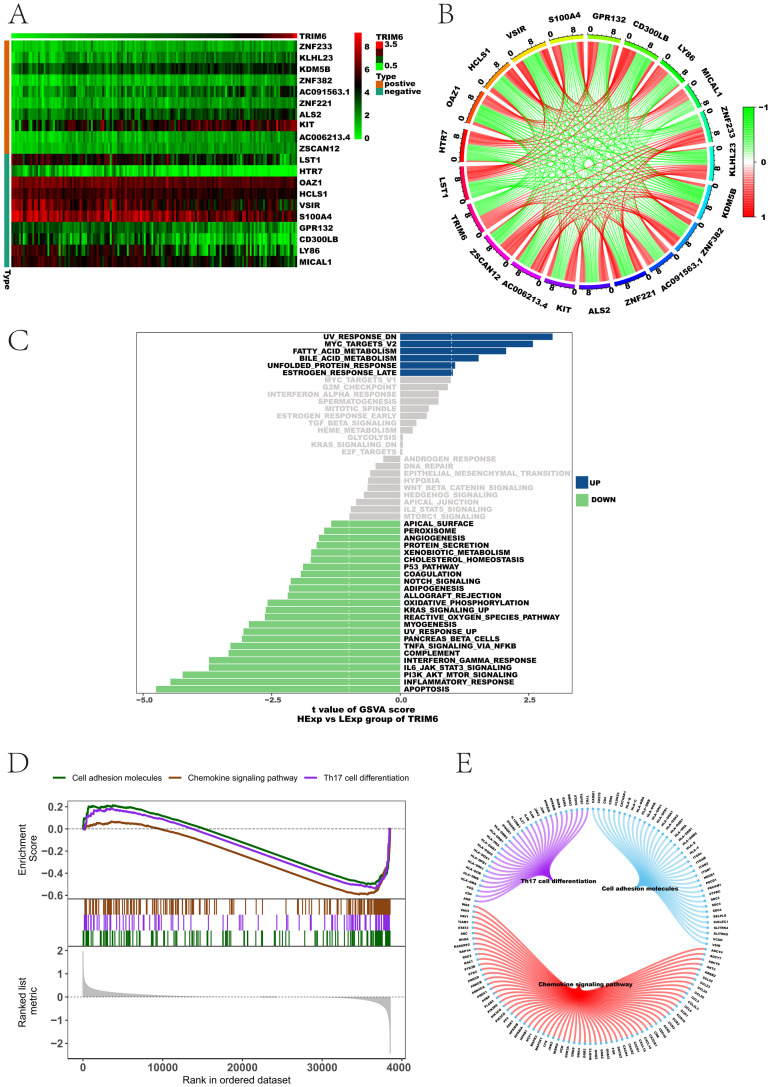
Enrichment analysis of genes and pathways related to TRIM6 in AML. (A) Heatmap of the top 10 genes with the highest positive/negative correlation with TRIM6 expression. (B) Co-expression correlation circle plot of the top 10 positively and negatively correlated genes with TRIM6, with red indicating positive correlation and green indicating negative correlation. (C) Signaling pathways involved with TRIM6 expression, with green indicating pathways affected by low TRIM6 expression. Background gene set: hallmark. (D-E) The signaling pathways and regulatory relationships involved in the TRIM6 gene.

From the TCGA database, 69 genes significantly correlated with TRIM6 expression were identified using correlation analysis (S1 Table), with a correlation coefficient threshold of 0.5 and a p-value cutoff of 0.05. The top 10 positively and negatively correlated genes were visualized in a heatmap (Fig 2A) and a co-expression correlation circle plot (Fig 2B). Genes with the highest positive correlation included ZSCAN12, AC006213.4, and KIT, while the top negatively correlated genes were OAZ1, HTR7, and LST1. To further explore the biological pathways implicated in AML pathogenesis, GSVA analysis was conducted. Enrichment analysis revealed associations with apoptosis, inflammatory response, and the PI3K/AKT/mTOR signaling pathway (Fig 2C). Additionally, GSEA results indicated connections with Th17 cell differentiation, cell adhesion molecules, and chemokine signaling pathways (FDR – adjusted p – value < 0.05, Fig 2D and 2E). These results suggest that TRIM6 may influence AML progression through these signaling pathways.

### 3.3 TRIM6 expression in patients with AML correlates with immune cell infiltration

**Fig 3:**
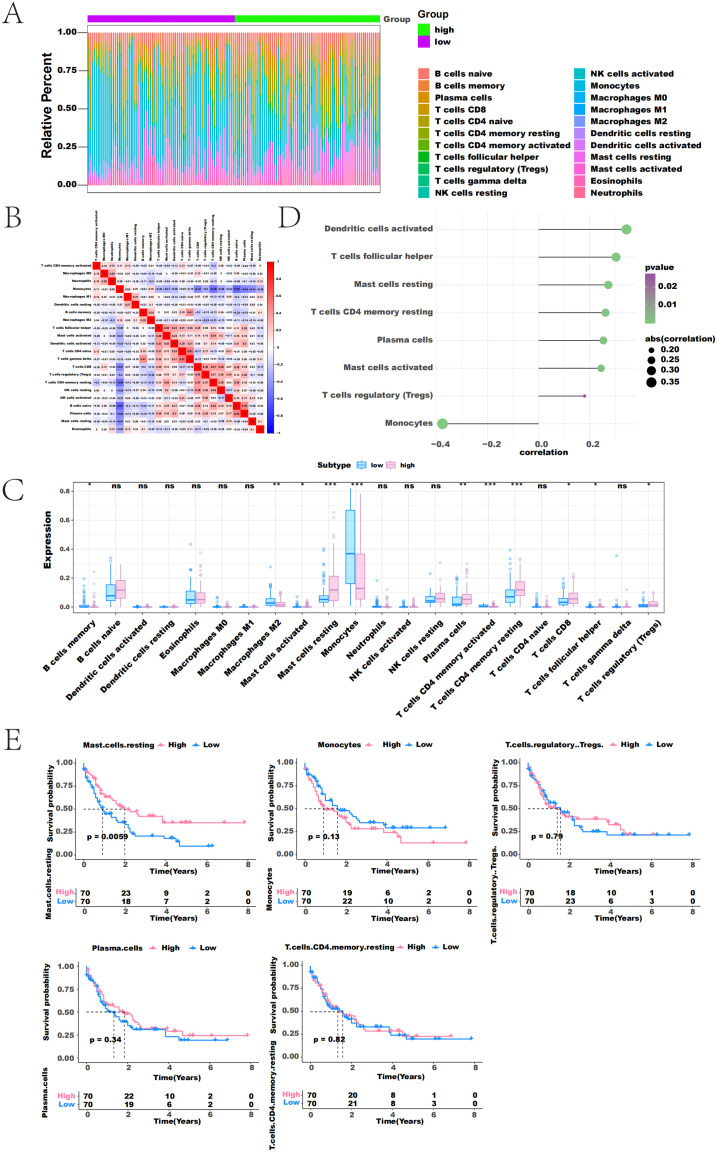
TRIM6 expression in patients with AML correlates with immune cell infiltration. (A) Relative percentages of immune cell subsets in patients with high and low TRIM6 expression. (B) Heatmap showing the correlation between immune cells, with blue indicating negative correlation and red indicating positive correlation. (C) Box-and-whisker plot comparing CIBERSORT analysis results between high and low TRIM6 expression groups. (D) Lollipop plot showing the correlation between immune cell infiltration levels and TRIM6 expression. (E) Survival analysis of immune cell infiltration levels in patients with AML.*p < 0.05, **p < 0.01, ***p < 0.001.

In this study, the TME and its impact on AML survival outcomes and clinical treatment sensitivity were explored. The relationship between TRIM6 expression and tumor immune cell infiltration was analyzed using data from the TCGA dataset. The results showed the proportion of immune cell content in each patient and the correlations between various immune cell types (Fig 3A and 3B). The “CIBERSORT” analysis indicated a significant correlation between TRIM6 expression and tumor-infiltrating immune cells. Several immune cell types showed significant differences between the high and low TRIM6 expression groups, including memory B cells, M2 macrophages, activated mast cells, resting mast cells, monocytes, plasma cells, activated memory CD4 + T cells, resting memory CD4 + T cells, CD8 + T cells, follicular helper T cells, and regulatory T cells (Fig 3C). In particular, the expression of TRIM6 was found to be significantly positively correlated with the abundance of various immune cells, including activated dendritic cells, follicular helper T cells, resting mast cells, resting memory CD4 + T cells, plasma cells, activated mast cells, and regulatory T cells. Conversely, TRIM6 showed a significant negative correlation with monocytes (Fig 3D). Notably, since the levels of activated dendritic cells, follicular helper T cells, and activated mast cell-infiltrating cells were almost zero in most samples, the study focused on the relationship between the infiltration levels of the remaining five immune cells and the prognosis of patients with AML. The findings revealed that lower levels of resting mast cell infiltration, defined by infiltration scores below the median, were associated with poor prognosis in patients with AML (Fig 3E). These results suggest that the expression of TRIM6 in AML correlates with immune cell infiltration. Furthermore, the association between resting mast cell infiltration and prognosis highlights that low TRIM6 expression may be a predictor of poor prognosis in patients with AML.

### 3.4 Mutation characterization and TRIM6 reverse-predicted miRNAs in patients with AML

**Fig 4:**
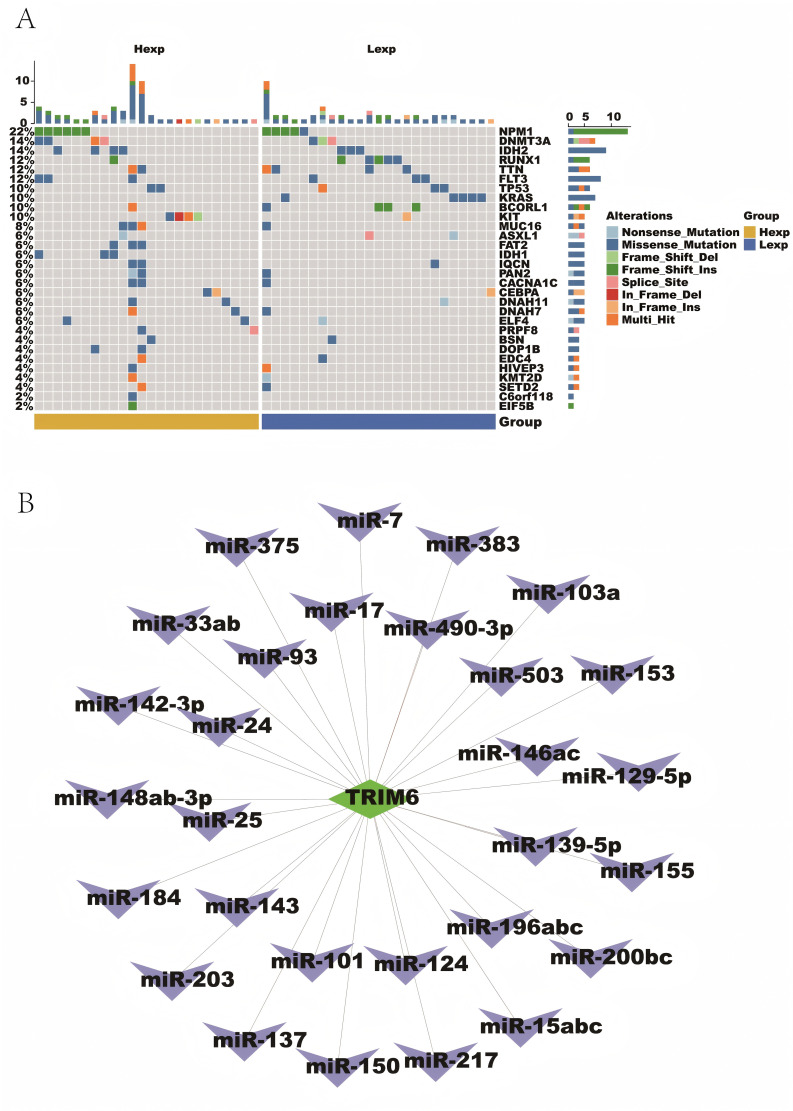
Mutational features and TRIM6-regulated miRNAs in patients with AML. (A) SNP data for AML, displaying the top 30 genes with the highest mutation frequency to compare gene mutations between the high and low expression patient groups. (B) TRIM6-mediated prediction of 29 miRNAs, with green indicating mRNAs and blue indicating miRNAs.

To analyze the mutation characteristics of patients with AML, processed Single Nucleotide Polymorphism (SNP) data related to AML were downloaded, and the TOP30 genes with the highest mutation frequencies were selected for comparison between patients with high and low TRIM6 expression. The mutation landscape was visualized using the ComplexHeatmap package in R (Fig 4A), which revealed differences in the mutated genes between these two groups. The analysis showed that in the low TRIM6 expression group, the percentage of mutated NPM1 genes was lower compared to the high TRIM6 expression group. On the other hand, genes such as RUNX1, TP53, and ASXL1 had higher mutation frequencies in the low TRIM6 expression group than in the high TRIM6 expression group. According to the European Leukemia Network Risk Stratification 2022 (ELN 2022) [23], NPM1 mutations (without FLT3-ITD mutations) are associated with a good prognosis, whereas mutations in RUNX1, TP53, and ASXL1 are linked to poor prognosis. This suggests that low TRIM6 expression may be indicative of a worse prognosis for patients with AML.

To further explore the regulatory mechanisms of TRIM6, reverse prediction was performed using the miRcode database, which identified 29 potential miRNA–mRNA interaction pairs. This suggests that TRIM6 may be subject to post-transcriptional regulation by multiple miRNAs. These interaction pairs were visualized using Cytoscape software, where nodes and edges intuitively represent the potential relationships between TRIM6 and the corresponding miRNAs (Fig 4B). This analysis expands the understanding of TRIM6 regulation and provides valuable clues for future experimental and functional studies, contributing to the elucidation of the biological role of the miRNA–TRIM6 axis.

### 3.5 TRIM6 expression and chemotherapy drug sensitivity analysis in patients with AML

**Fig 5:**
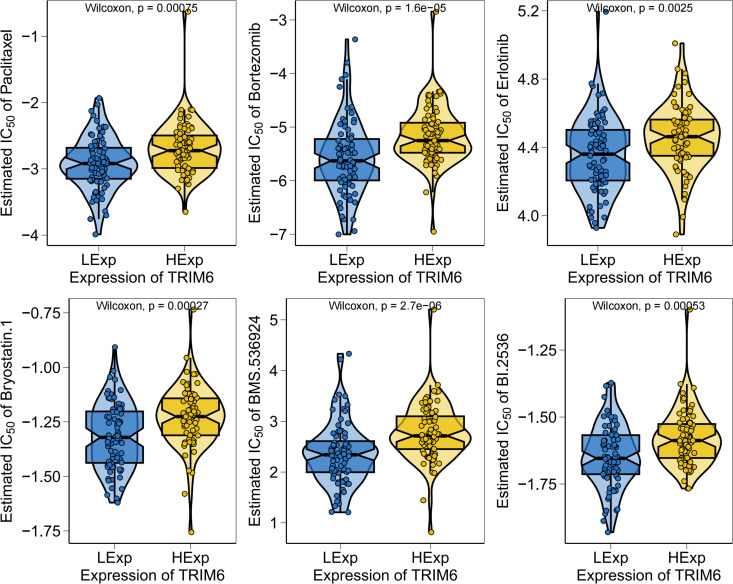
TRIM6 expression in patients with AML exhibiting chemotherapeutic drug sensitivity.

Chemotherapy remains a cornerstone in the treatment of AML. This study leveraged drug sensitivity data from the GDSC database to predict chemotherapy sensitivity in tumor samples using the R package “pRRophetic.” The objective was to explore the relationship between TRIM6 gene expression and the sensitivity to commonly used chemotherapeutic agents. Differential half inhibitory concentration (IC50) scores for chemotherapeutic drugs, including paclitaxel, bortezomib, and erlotinib, were compared between TRIM6 high- and low-expression groups. Results indicated that patients in the low-expression group exhibited significantly lower IC50 values for paclitaxel, bortezomib, and erlotinib, compared to the high-expression group (Fig 5). Paclitaxel has been shown to inhibit proliferation and promote apoptosis of FLT3-ITD-mutated MV4–11 AML cells by suppressing the PI3K/AKT/mTOR signaling pathway [24].Bortezomib has been shown to display synergistic anti-AML cytotoxicity in vitro when combined with low-dose decitabine, Furthermore, the erlotinib complex exhibits anti-AML potential by inducing dendritic cell differentiation of leukemia cells and remodeling the immunosuppressive microenvironment [25], thereby suggesting heightened therapeutic sensitivity in the low-expression group. Notably, no significant differences were observed between high and low TRIM6 expression for other common AML chemotherapeutics, such as all-trans retinoic acid, cytarabine, and cisplatin (S1 Fig). These results highlight the correlation between TRIM6 expression levels and sensitivity to specific chemotherapy regimens, positioning TRIM6 as a potential biomarker for personalized AML therapy.

### 3.6 TRIM6 exhibits prognostic significance in patients with AML

**Fig 6:**
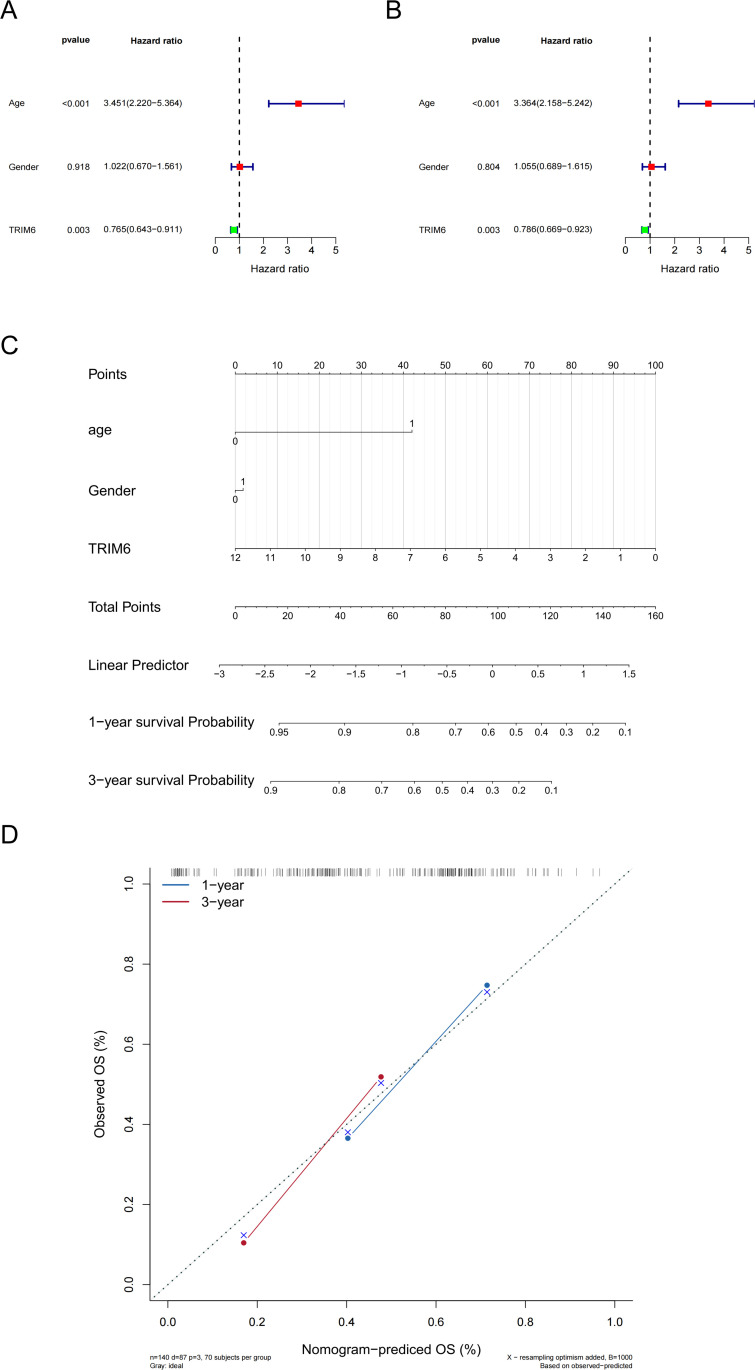
TRIM6 exhibits prognostic significance in patients with AML. (A) One-way Cox regression analysis. (B) Multivariate Cox regression analysis. (C) Column-linear graphical model illustrating variable interrelationships. (D) Calibration curves for 1-year and 3-year OS predictions.

One-way Cox regression analysis was conducted to assess the relationship between TRIM6 expression and clinical characteristics, using the median expression as a threshold. The forest plot revealed a significant correlation between age (HR = 3.451, 95% CI: 2.220–5.364, p < 0.001) and TRIM6 expression (HR = 0.765, 95% CI: 0.643–0.911, p = 0.03) with OS (Fig 6A). Subsequent multivariate Cox regression analysis confirmed that both age (HR = 3.364, 95% CI: 2.158–5.242, p < 0.001) and TRIM6 (HR = 0.786, 95% CI: 0.669–0.923, p = 0.03) were independent prognostic factors for OS (Fig 6B). A prognostic line graph was then constructed, showing that higher scores were associated with poorer prognosis. These results suggest that TRIM6 expression could serve as a reliable prognostic indicator for AML (Fig 6C). Furthermore, the calibration curve model validated TRIM6 as an accurate predictor for 1-year and 3-year survival outcomes, reinforcing its potential as a prognostic biomarker (Fig 6D). In conclusion, TRIM6 may function as a tumor-suppressive factor in AML and holds significant prognostic value.

### 3.7 Overexpression of TRIM6 inhibits the PI3K/AKT pathway affecting AML progression

**Fig 7:**
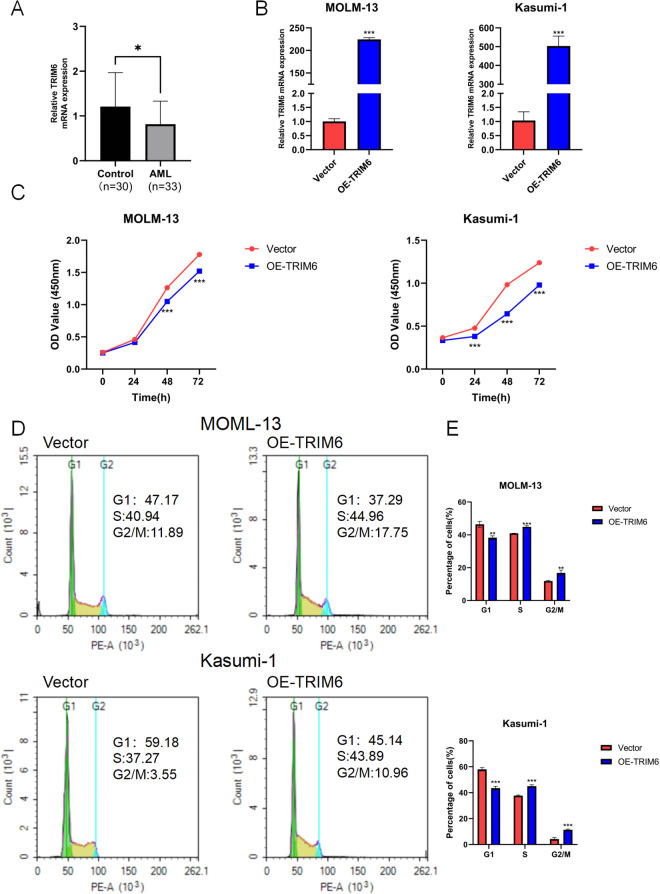
Overexpression of TRIM6 inhibits the PI3K/AKT pathway affecting AML progression. (A) qRT-PCR analysis of TRIM6 expression in patient and normal human samples. (B) qRT-PCR confirmation of TRIM6 overexpression in MOLM-13 and Kasumi-1 cells. (C) CCK-8 assay to assess proliferation in TRIM6-overexpressing MOLM-13 and Kasumi-1 cells. (D) Flow cytometry analysis of cell cycle distribution in MOLM-13 and Kasumi-1 cells after TRIM6 overexpression. (E) Statistical analysis of (D).*p < 0.05, **p < 0.01, ***p < 0.001.

**Fig 8:**
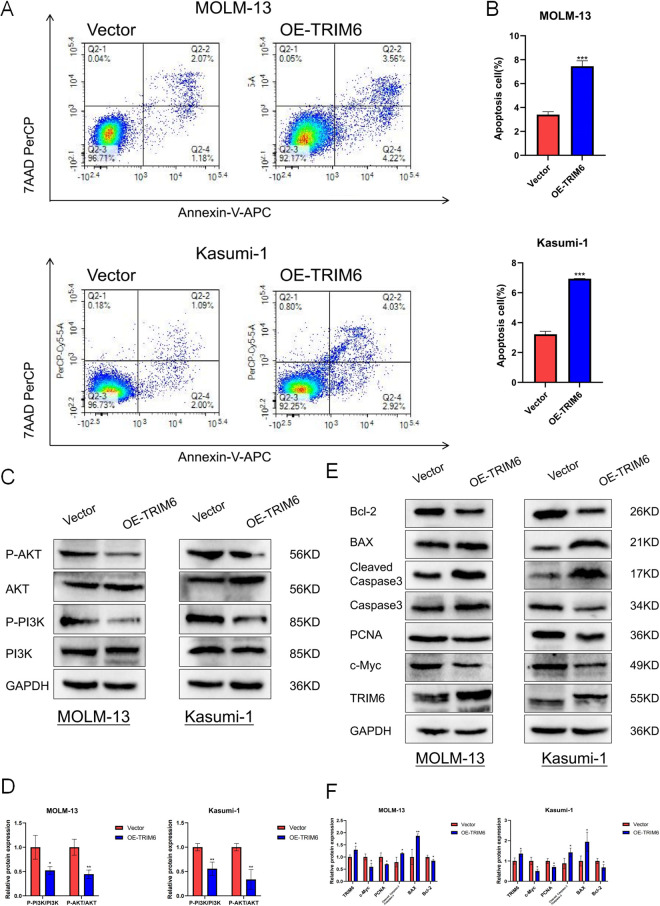
(A) Apoptosis rate of MOLM-13 and Kasumi-1 cells after TRIM6 overexpression, detected by flow cytometry. (B) Statistical analysis of (A). (C) Western blot analysis of AKT, P-AKT, PI3K, and P-PI3K protein expression. (D) Statistical analysis of (C). (E) Western blot analysis of TRIM6, c-Myc, PCNA, Caspase 3, Cleaved Caspase 3, Bax, and Bcl-2 protein expression. (F) Statistical analysis of (E).*p < 0.05, **p < 0.01, ***p < 0.001.

To elucidate the specific role of TRIM6 in AML development, 33 AML patient samples and 30 healthy controls were analyzed by qRT-PCR to assess TRIM6 expression levels. The results demonstrated significantly lower TRIM6 expression in patients with AML compared to healthy individuals (Fig 7A), consistent with bioinformatics analysis. MOLM-13 and Kasumi-1 cells stably overexpressing TRIM6 were generated using lentiviral vectors, and overexpression efficiency was confirmed by qRT-PCR and Western blot (Figs 7B and 8A). CCK-8 assays showed reduced proliferation in the OE-TRIM6 groups of both MOLM-13 and Kasumi-1 cells at 48 and 72 hours, compared to the vector control group (Fig 7C), indicating that TRIM6 inhibits AML cell proliferation. Flow cytometry analysis of cell cycle distribution revealed a significant reduction in the proportion of cells in the G1 phase in the OE-TRIM6 group, with a corresponding increase in the S and G2/M phases (Fig 7D and 7E), suggesting that TRIM6 may regulate cell cycle progression. Additionally, apoptosis rates were significantly elevated in both MOLM-13 and Kasumi-1 cells in the OE-TRIM6 group compared to the control group, as detected by flow cytometry (Fig 8A and 8B), indicating that TRIM6 induces apoptosis in AML cells. Together, these results suggest that TRIM6 exerts an oncogenic effect in AML by inhibiting cell proliferation, modulating cell cycle progression, and promoting apoptosis.

GSVA indicated that the PI3K/AKT signaling pathway is crucial in the pathological process of AML. This pathway has been extensively studied for its role in regulating cell proliferation and apoptosis and is considered a fundamental molecular mechanism driving AML progression [26,27]. To further investigate the impact of TRIM6 on PI3K and AKT phosphorylation in AML, the phosphorylation levels of PI3K and AKT were examined in MOLM-13 and Kasumi-1 cells. Western blot analysis revealed significantly reduced levels of P-PI3K and P-AKT in the OE-TRIM6 group compared to the Vector control (Fig 8C and 8D). Additionally, increased expression of Bax and Cleaved Caspase 3 proteins, along with decreased expression of Bcl-2, in the OE-TRIM6 group confirmed that TRIM6 promotes apoptosis in AML cells. Furthermore, the expression of PCNA and c-Myc proteins was lower in the OE-TRIM6 group than in the control group, suggesting that TRIM6 inhibits AML cell proliferation (Fig 8E and 8F). These results collectively suggest that TRIM6 overexpression modulates AML cell proliferation and apoptosis by inhibiting the PI3K/AKT signaling pathway, thereby affecting the progression of AML.

## 4. Discussion

AML is one of the most prevalent and fatal cancers globally. Despite significant advancements in treatment, the 5-year OS rate for patients with relapsed or refractory AML, or those ineligible for hematopoietic stem cell transplantation (HSCT), remains disappointingly low [28]. Statistically, only approximately 20% of patients with elderly AML survive for two years after diagnosis [5]. Given the current landscape of AML treatment, refining precision risk stratification and prognostic management represents a critical approach to improving clinical outcomes. Therefore, the identification of reliable biomarkers for prognostication and guiding personalized therapy is of paramount importance. In this study, a dataset was utilized to identify the TRIM6 gene as a potential prognostic marker to explore its mechanisms in AML.

TRIM6 is highly expressed in various cancers and exerts oncogenic functions. For example, in breast cancer, TRIM6 promotes tumor progression by facilitating the degradation of STUB1, thereby enhancing cancer cell proliferation and migration [29]; in colorectal cancer, TRIM6 promotes tumor cell proliferation through regulation of the TIS21/FoxM1 axis [30]; while in glioma, TRIM6 drives malignant progression by promoting the ubiquitination and degradation of FOXO3A [31]. In contrast, in AML studied here, TRIM6 exhibits potential tumor-suppressive effects, with its downregulation closely associated with poor prognosis. This may be related to its negative regulation of the PI3K/AKT signaling pathway, inducing apoptosis and inhibiting cell proliferation. Such functional differences may stem from variations in the types of target proteins mediated by TRIM6 in different cellular environments, the composition of regulatory networks, and the degree of its ubiquitination modification. Therefore, TRIM6 does not exhibit a singular function across tumor types but displays a context-dependent dual role.

Analysis of the TCGA-AML cohort revealed low TRIM6 expression in patients with AML, and both univariate and multivariate Cox regression analyses identified low TRIM6 expression as a poor prognostic factor for survival. Further investigation showed that the frequency of NPM1 mutations was lower in the low TRIM6 expression group, while mutations in high-risk genes such as RUNX1, TP53, and ASXL1 were more frequent. According to the ELN 2022 risk stratification criteria, NPM1 mutations (in the absence of FLT3-ITD co-mutations) are considered favorable, while RUNX1, TP53, and ASXL1 mutations are associated with poor prognosis [23]. This further substantiates the notion that reduced TRIM6 expression negatively impacts the survival of patients with AML. Taken together, low TRIM6 expression emerges as a potential adverse prognostic marker for AML patients.Building upon these findings, we further explored the potential regulatory miRNAs associated with TRIM6. In addition to their possible roles in AML, several predicted miRNAs targeting TRIM6 have been reported to possess prognostic significance in other malignancies, indicating their broader biological relevance. For instance, miR-139-5p, which was identified in the prediction, has been shown to have prognostic value in various cancers [32], including osteosarcoma [33], glioma [34], and bladder cancer. Another miRNA, miR-490-3p, was found to be downregulated in colorectal cancer, and its low expression is associated with poor prognosis. This miRNA promotes cell proliferation and invasion by targeting RAB14 [35].These findings suggest that the TRIM6-related miRNAs identified in this study may not only play functional roles in AML but also warrant further investigation in the context of other cancer types.

The TME, composed of tumor cells, fibroblasts, immune cells, endothelial cells, mesenchymal stem cells, and adipocytes [36]. It forms a dynamic equilibrium environment for host immune surveillance and tumor immune escape through the continuous secretion of cytokines, growth factors, and chemokines [36,37]. TME plays a crucial role in tumor development and has significant prognostic value for AML [38–40]. Using the CIBERSORT algorithm, TRIM6 expression was found to be significantly associated with resting mast cells, monocytes, resting memory CD4 + T cells, and M2 macrophages. In the low-risk group, higher proportions of resting memory CD4 + T cells and resting mast cells were observed. Median survival analysis indicated that low resting mast cell infiltration predicted poor prognosis. In the high-risk group, increased infiltration of monocytes and M2 macrophages was noted, consistent with studies identifying these cells as negative prognostic indicators in prostate and gastric cancers [41,42]. These results suggest a strong link between immune cell infiltration and adverse outcomes in AML, with TRIM6 expression closely related to immune landscape alterations.Nevertheless, the findings are based on transcriptomic data and computational estimation, reflecting associations rather than causality. The functional state and dynamic changes of immune cells remain unaddressed. Further in vivo and in vitro validation is needed to clarify the mechanistic role of TRIM6 in modulating immune cell infiltration and function in AML.

Cancer development is a highly complex process involving multiple molecular and cellular abnormalities. During this process, the PI3K/AKT signaling pathway is frequently dysregulated, serving as a critical regulatory node in the development of various cancers. Inhibition of the PI3K/AKT pathway has been shown to effectively suppress the progression of cancers such as breast cancer [43], small cell lung cancer [44], and cervical cancer [45]. Numerous studies have highlighted the essential role of the PI3K/AKT signaling pathway in regulating the proliferation, growth, invasion, metabolism, and motility of cancer cells [27]. Specifically, it has been demonstrated that RBM39 promotes AML cell proliferation and inhibits apoptosis through activation of the PI3K/AKT pathway [46]. In our study, GSEA results revealed that AML development may also be associated with pathways such as Cell Adhesion Molecules, Chemokine Signaling, and Th17 Cell Differentiation, suggesting additional molecular mechanisms potentially involved in AML progression. Cell Adhesion Molecules, a group of cell surface proteins [47], mediate cell-to-cell or cell-to-extracellular matrix interactions and are involved in various cellular processes, including adhesion, recognition, signaling, and the regulation of cell proliferation [48–50]. Chemokines play a pivotal role in cancer progression by contributing to immune evasion, tumor growth, and metastasis [51]. Th17 cells, a unique subset of proinflammatory CD4 + T helper cells, are critical in mediating inflammatory responses to infections and are also implicated in the pathogenesis of autoimmune diseases and cancer [52]. Our findings highlight that the interplay between these immune-related pathways and cell types may play a previously underappreciated role in AML pathogenesis, providing new insights into its molecular landscape and offering potential avenues for therapeutic intervention.

**Fig 9:**
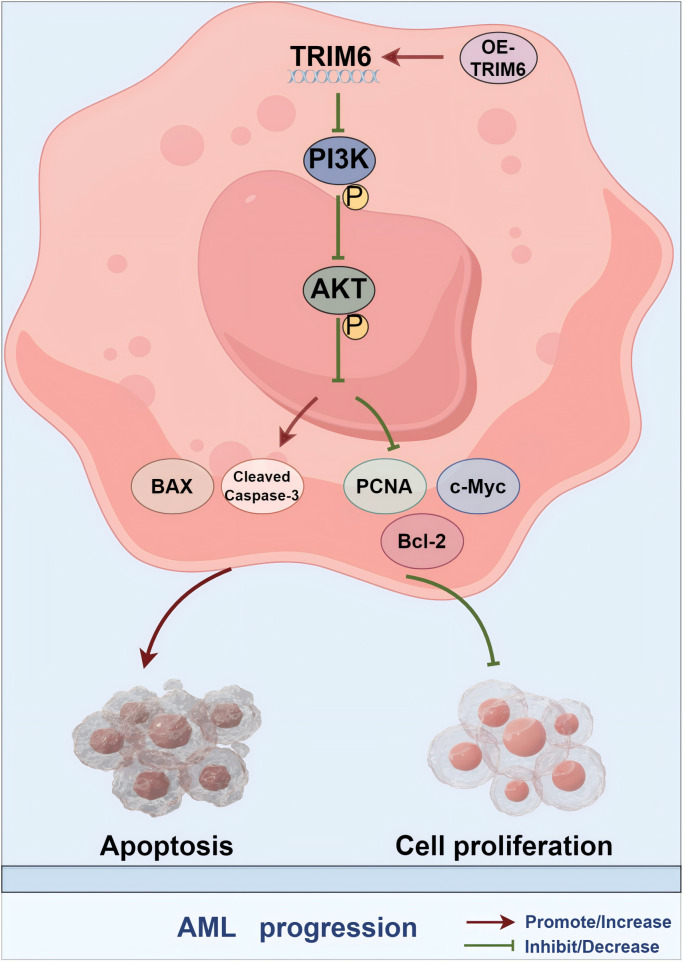
Schematic model of the role of the TRIM6-PI3K/AKT axis in AML.

This study further confirmed the abnormal downregulation of TRIM6 gene expression in patients with AML through qRT-PCR analysis of peripheral blood samples from 33 patients with AML and 30 healthy controls.However, it should be noted that the relatively small sample size may limit the statistical power and robustness of the analysis. Therefore, future studies with larger and independent patient cohorts are necessary to validate these findings and strengthen the reliability and generalizability of the conclusions. The CCK-8 assay and flow cytometry results demonstrated that TRIM6 overexpression in MOLM-13 and Kasumi-1 AML cells inhibited cell proliferation, altered cell cycle progression, and promoted apoptosis. Previous studies have shown that silencing PROK1 exerts its anti-pancreatic cancer effects by inhibiting the PI3K/AKT/mTOR pathway, leading to increased levels of Bax and Cleaved Caspase 3, and decreased levels of PCNA and Bcl-2 in pancreatic cancer cells [53]. Similarly, STIL has been reported to decrease c-Myc expression *via* the PI3K/AKT/mTOR pathway, thereby inhibiting bladder cancer cell proliferation [54]. These findings are consistent with the present study, where TRIM6 overexpression increased Bax and Cleaved Caspase 3 protein levels in AML cells, while Bcl-2, PCNA, c-Myc, P-PI3K, and P-AKT protein levels were significantly reduced, as shown by Western blot analysis.However, in vivo experiments have not yet been conducted to further validate these findings. In future studies, we plan to establish xenograft mouse models to observe the effects of TRIM6 overexpression on tumor growth and disease progression, thereby enabling a more comprehensive evaluation of the therapeutic potential of TRIM6 in the context of AML. In summary, both database analyses and experimental results robustly demonstrate that TRIM6 overexpression inhibits AML cell proliferation and induces apoptosis through the PI3K/AKT signaling pathway, as illustrated in Fig 9 (created using Figdraw).

## 5. Conclusion

In conclusion, low TRIM6 expression in patients with AML is closely associated with poor overall survival, potentially linked to immune cell infiltration and the regulation of the PI3K/AKT signaling pathway, and is involved in proliferation, apoptosis, and cell cycle processes, making it a potentially important prognostic biomarker for AML. However, this study has some limitations, including the lack of in vivo validation, a relatively small clinical validation cohort, and an incomplete understanding of how TRIM6 mediates tumor–immune microenvironment interactions. Future research should address these gaps to better elucidate TRIM6’s mechanisms and assess its potential clinical utility in AML diagnosis and therapy.

## Supporting information

S1 Table Genes significantly correlated with TRIM6 expression in TCGA AML cohort. (XLSX)

S1 Fig Absence of TRIM6 expression-associated sensitivity to ATRA, cytarabine and cisplatin in AML. (PDF)

S1 Raw images Original uncropped Western blot images. (PDF)
