# Supplementary material for: TRIM6 affects the prognosis of acute myeloid leukemia through the PI3K/AKT signaling pathway and is associated with immune infiltration
Source: PLoS One. 2025 Sep 17;20(9):e0329560. doi: 10.1371/journal.pone.0329560 (PMC12443272; doi:10.1371/journal.pone.0329560)

FIG8-C-MOLM13

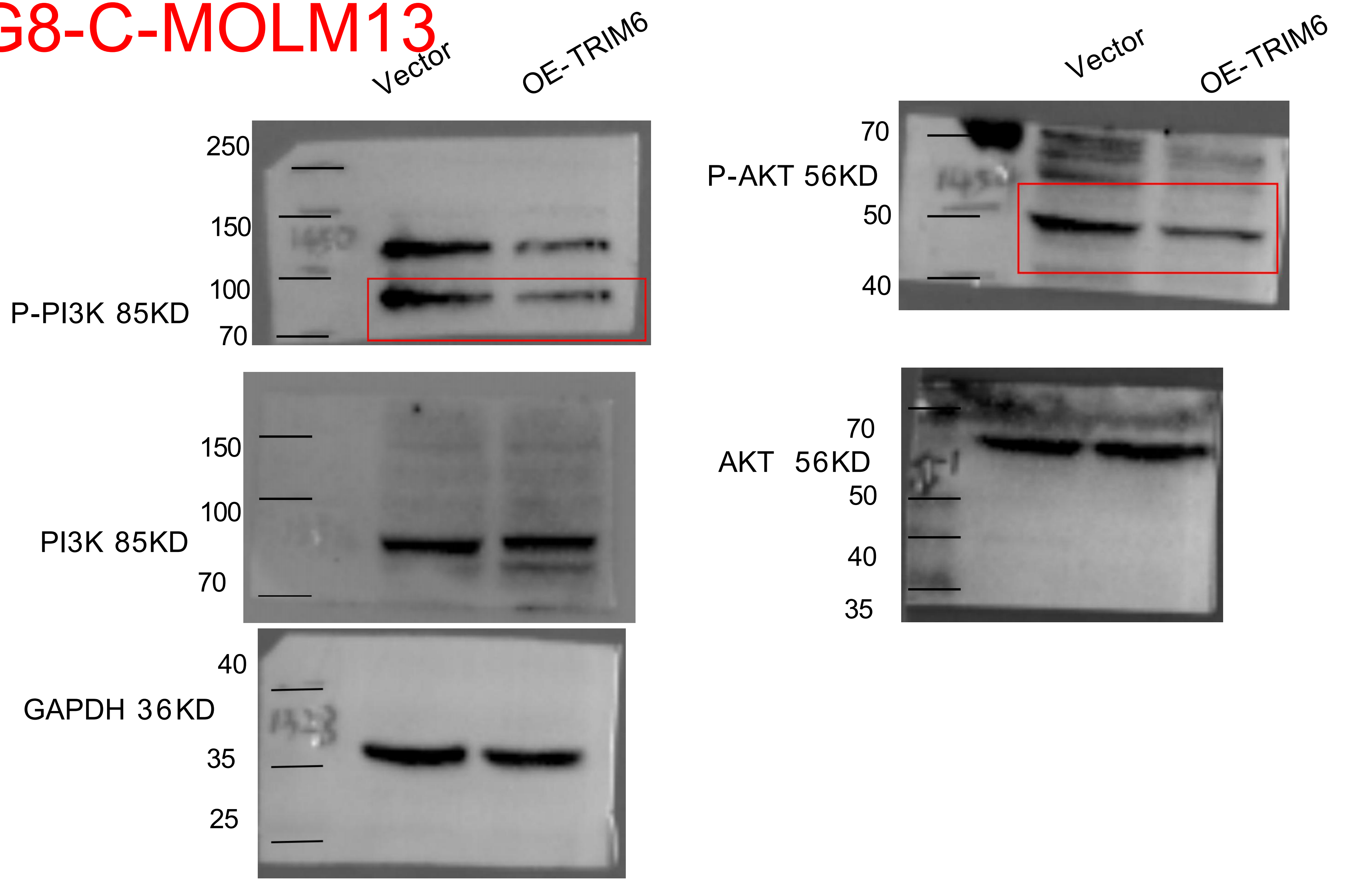

MOLM13

FIG 8 -C -Kasumi -1

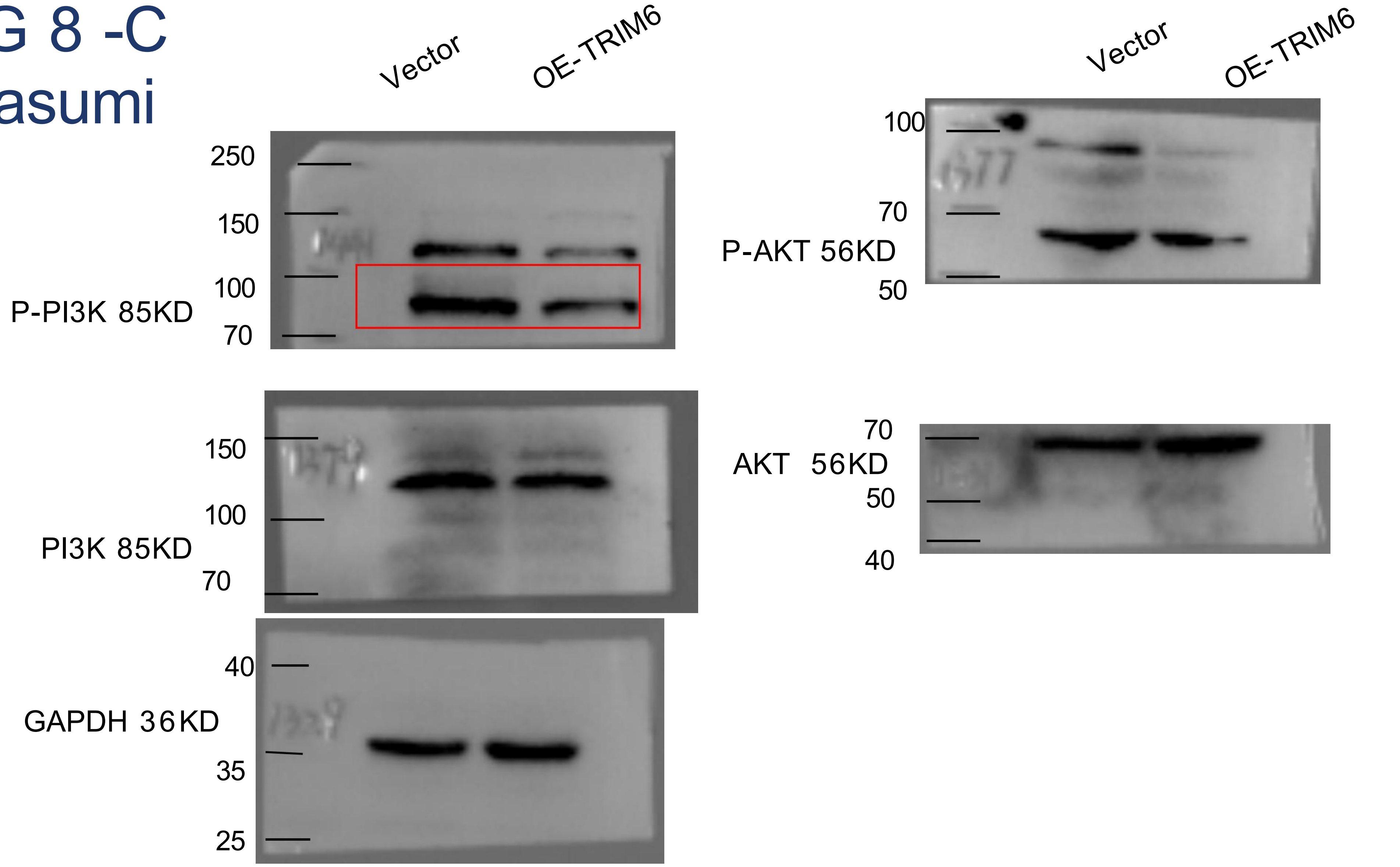

Kasumi-1

FIG8-E-MOLM13

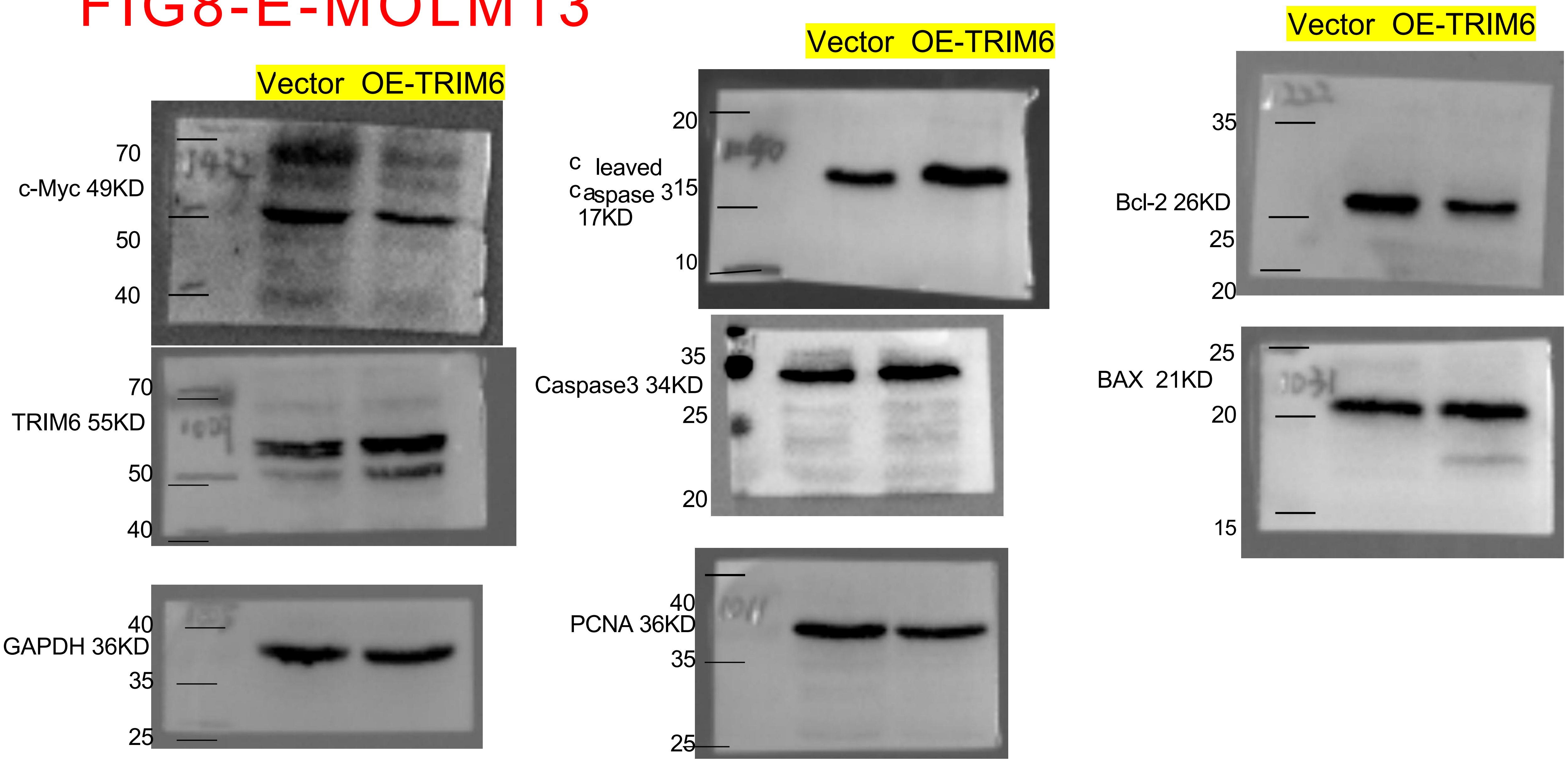

FIG8-E-Kasumi-1

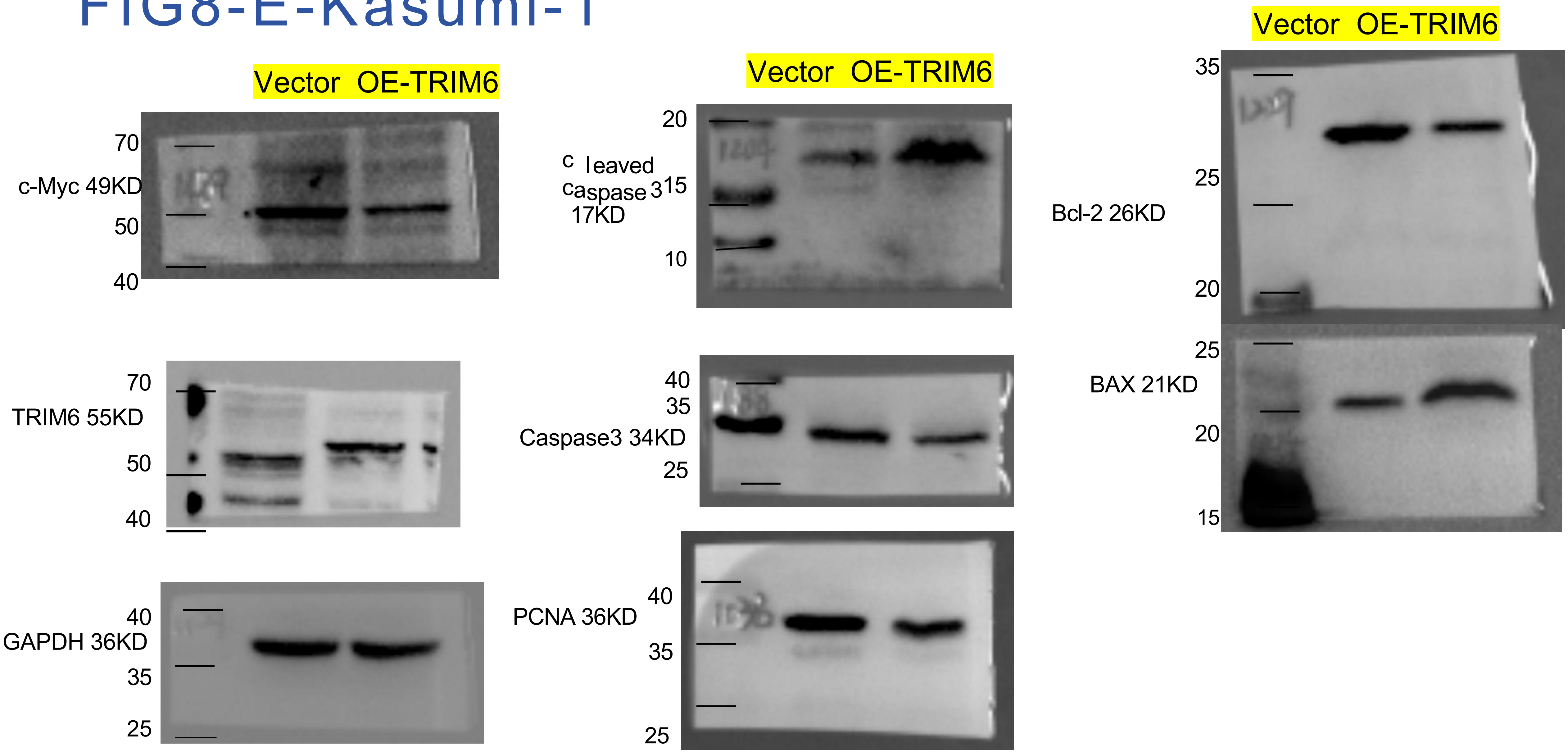

Supplement: S1 Raw images — (PDF) [file pone.0329560.s003.pdf]
